# EpisomiR, a New Family of miRNAs, and Its Possible Roles in Human Diseases

**DOI:** 10.3390/biomedicines10061280

**Published:** 2022-05-30

**Authors:** Yasuko Arao, Mika Nakayama, Yoshiko Tsuji, Yumiko Hamano, Chihiro Otsuka, Andrea Vecchione, Ken Ofusa, Hideshi Ishii

**Affiliations:** 1 Center of Medical Innovation and Translational Research, Department of Medical Data Science, Osaka University Graduate School of Medicine, Yamadaoka 2-2, Suita 565-0871, Osaka, Japan; arao@cfs.med.osaka-u.ac.jp (Y.A.); nakayama@cfs.med.osaka-u.ac.jp (M.N.); tsuji@cfs.med.osaka-u.ac.jp (Y.T.); hamano@cfs.med.osaka-u.ac.jp (Y.H.); cot@cfs.med.osaka-u.ac.jp (C.O.); oof21443@ideacon.co.jp (K.O.); 2 Department of Clinical and Molecular Medicine, Santo Andrea Hospital, University of Rome “Sapienza”, Via di Grottarossa, 1035-00189 Rome, Italy; andrea.vecchione@uniroma1.it; 3 Food and Life-Science Laboratory, Prophoenix Division, IDEA Consultants, Inc., Osaka 559-8519, Osaka, Japan

**Keywords:** miRNA variants, methylation, RNA modification

## Abstract

MicroRNAs (miRNAs) are synthesized through a canonical pathway and play a role in human diseases, such as cancers and cardiovascular, neurodegenerative, psychiatric, and chronic inflammatory diseases. The development of sequencing technologies has enabled the identification of variations in noncoding miRNAs. These miRNA variants, called isomiRs, are generated through a non-canonical pathway, by several enzymes that alter the length and sequence of miRNAs. The isomiR family is, now, expanding further to include episomiRs, which are miRNAs with different modifications. Since recent findings have shown that isomiRs reflect the cell-specific biological function of miRNAs, knowledge about episomiRs and isomiRs can, possibly, contribute to the optimization of diagnosis and therapeutic technology for precision medicine.

## 1. Introduction

The central dogma of molecular biology describes a biochemical flow from genomic DNA to the transcriptome. Peptide-coding messenger RNAs (mRNAs) are, then, translated to proteins, whereas regulatory RNAs are involved in various processes. For example, noncoding RNAs, including microRNAs (miRNAs), which are approximately 22 base pairs in length, regulate posttranscriptional mechanisms [1].

Although the genome encodes miRNAs, Morin et al., demonstrated that isomiRs, which are variants of reference miRNA sequences, are expressed in human embryonic stem cells and are implicated in cellular differentiation, cell cycle control, programmed cell death, and transcriptional regulation [2], in a way similar to the reference miRNA sequences [3,4]. The gold standard of DNA and RNA analyses is the comparison of sequences obtained using Sanger’s method [5], or more recently, the Illumina sequencing method [6], with reference sequences. Additionally, expression analysis of particular sequences can be performed using microarray or chromatin immunoprecipitation assays, in which a large number of samples (usually 1000 or more) are analyzed, simultaneously [7]. These methods are useful for investigating the expression of target nucleotides, given that the same miRNA precursor generates the same miRNA sequence. However, recent studies using deep sequencing analysis, rather than a comparison with reference sequences, have identified tremendous variability in the biogenesis of miRNAs, which was previously uncharacterized. Interestingly, these studies suggest that the same miRNA precursor may generate many different sequences that might have different targets and effects on mRNAs [8,9,10], which contradicts previously proposed findings [8]. The investigation of isomiRs could reveal uncharacterized features of miRNAs and provide valuable insight into the understanding, detection, and therapeutic applications of nucleotides. In this study, we searched the PubMed database (Available online: https://pubmed.ncbi.nlm.nih.gov, accessed on 28 March 2022), using the following search terms listed, up to 2022: miRNA, isomer, RNA variation, RNA modification, and RNA methylation.

## 2. Regulatory miRNAs Are Implicated in Human Diseases

Fire and Mellow, together with their colleagues, studied the translation of small double-stranded RNAs (dsRNAs) into proteins, by disrupting mRNAs with complementary sequences. They reported that dsRNA suppresses gene expression more efficiently than single-stranded RNA, and that a catalytic process was involved [11,12]. Other studies investigating the developmental timing pathway of *Caenorhabditis elegans* identified the miRNAs lin-4 and lin-14, by genetic analyses in developmental timing mutants [13,14]. This pathway was evidenced by the pioneering work of Sulston and Horvitz [15,16].

### 2.1. Canonical Pathway for Generating miRNAs

**Figure 1:**
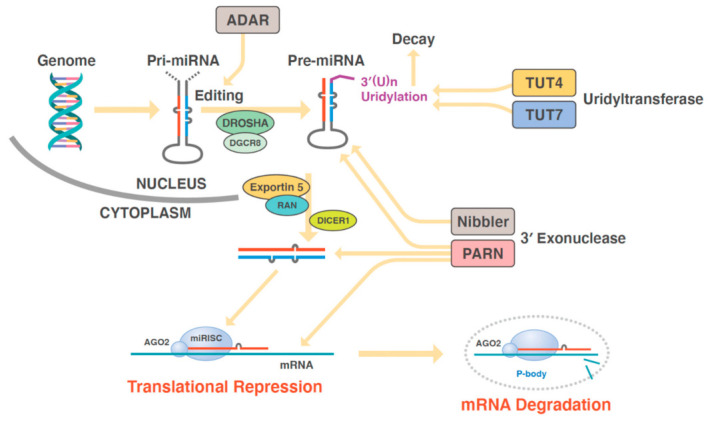
Synthesis of miRNAs from the genome. Enzymes involved in the synthesis of miRNAs are: ADAR, adenosine deaminase RNA specific; AGO2, argonaute RISC catalytic component 2; DICER1, dicer 1, ribonuclease III; DGCR8, DiGeorge syndrome critical region gene 8, DGCR8 microprocessor complex subunit; DROSHA, drosha ribonuclease III; miRISC, miRNA-induced silencing complex; Nibbler Homolog, EXD3, exonuclease 3′–5′ domain containing 3; PARN, poly(A)-specific ribonuclease; RAN, a member of RAS oncogene family; TUT4, terminal uridylyltransferase 4; and TUT7, terminal uridylyltransferase 7.

RNA polymerase II transcribes the sequences of genomic regions generating miRNAs into single-stranded RNAs. Then, the complementary portions are endogenously added to form dsRNAs [17]. These dsRNAs adopt a final hairpin loop structure, i.e., a shape similar to that of transfer RNAs (tRNAs) [18], and are called primary miRNAs (pri-miRNAs) [19]. Pri-miRNAs contain several hundred to several thousand bases, with a cap structure on the 5′-terminus (5′-cap) formed as 7 mGppp, i.e., 7-methyl guanosine and a polyadenosine tail on the 3′-terminus or 3′-poly(A) tail [20] (Figure 1).

An RNase III-like enzyme called DROSHA, which is present in the nucleus, cleaves pri-miRNA molecules to form precursor miRNAs (pre-miRNAs) that have a stem-loop structure and a length of approximately 70 bases [21]. The pre-miRNA molecules are, then, exported from the cell nucleus by a carrier protein called exportin-5 [22]. After their release into the cytoplasm, the pre-miRNAs are processed or spliced by an enzyme called DICER, thereby generating 20–25-base-long double-stranded miRNAs [23]. Double-stranded miRNAs are, subsequently, incorporated into the RNA-induced silencing complex (RISC), consisting of argonaute (AGO) proteins [24,25]. Each double-stranded miRNA incorporated into the RISC is processed into two single-stranded miRNAs, and the more unstable of these two is degraded [26]. The stable single-stranded miRNA has a sequence that is partially complementary to the 3′-untranslated region (UTR) of mRNAs; it can, thus, bind to such mRNAs and inhibit gene translation [27]. Such single-stranded miRNAs are called mature miRNAs or functional miRNAs [28].

The region spanning 2nd–8th base on the 5′-terminus of mature miRNAs is called the seed sequence [29], and mRNAs with sequences complementary to the seed sequence are strongly recognized as targets for gene silencing [30]. It was indicated that mature miRNAs and mRNAs bind according to the Watson–Crick base pairing. Consequently, mature miRNAs with GC-rich seed sequences stably bind to the 3′-UTR of mRNAs [31].

### 2.2. Implication of miRNAs in Human Diseases

Evidence supports the involvement of miRNAs in the occurrence and progression of several human diseases, such as cardiovascular [32], neurodegenerative [33], psychiatric [34], and chronic inflammatory [35,36] diseases as well as cancer [37].

#### 2.2.1. Involvement of miRNAs in Human Cancers

The miRNAs miR-15 and miR-16 were first identified in humans, by the positional cloning of tumor suppressor genes on chromosome 13, to investigate hematopoietic malignancies [38]. The expression of miR-15 and miR-16 has been associated with the prognosis and progression of chronic lymphocytic leukemia [39]. Additionally, the expression of the miRNA let-7 is reduced in human solid tumors, such as lung cancer, and is associated with shorter postoperative survival [40]. Moreover, while the use of mRNA expression profiles for the classification of tumors yields highly inaccurate results, the use of miRNA expression profiles can, successfully, classify poorly differentiated tumors, indicating its potential in cancer diagnosis [41].

#### 2.2.2. Involvement of miRNAs in Human Cardiovascular Diseases

The potential of miRNAs as novel biomarkers for the diagnosis and prognosis of cardiovascular diseases has recently emerged. Indeed, miRNAs are detectable in the blood, and their expression levels reflect the condition of the cardiovascular system at a cellular level. In particular, they are indicators of angiogenesis, cardiac cell contractility, lipid metabolism, plaque formation, cardiac rhythm, and cardiac cell growth [42]. A previous report has indicated that miR-1 and miR-133, which are clustered on the same chromosomal loci, are cotranscribed in a tissue-specific manner during development [43]. These two miRNAs play distinct roles in modulating the proliferation of skeletal muscle cells and the differentiation of cultured myoblasts, wherein miR-1 is involved in the inhibition of histone deacetylase 4 (HDAC4), a transcriptional repressor of muscle gene expression, and miR-133 participates in the repression of serum response factor, resulting in increased myoblast proliferation. Thus, a fine-tuned control of miRNAs synthesis may contribute to the transcriptional regulation of skeletal muscle gene expression and embryonic development, and altering this regulatory mechanism has significant consequences involving multiple factors [43]. Recently published papers present detailed information about miRNA structure, biogenesis, functions, and expression profiles in human cardiovascular diseases [42,44,45]. These studies have focused on the mechanisms controlled by miRNAs in cardiovascular diseases, such as hypertension, tissue ischemia, cardiac remodeling, arrhythmias, and atherosclerosis.

#### 2.2.3. Involvement of miRNAs in Human Neurodegenerative Diseases

Given that oxidative stress induces neuronal and glial cell degeneration in the central and peripheral nervous systems and is, thus, implicated in several major neurodegenerative disorders, the identification of accurate biomarkers for monitoring oxidative stress has become, increasingly, important [46]. Oxidative stress affects the expression levels of multiple miRNAs, and miRNAs regulate the expression of many genes involved in the oxidative stress response. Thus, miRNA networks and oxidative stress could be, inextricably, linked in the neurodegenerative processes involved in Alzheimer’s disease, Parkinson’s disease, Huntington’s disease, and amyotrophic lateral sclerosis [46]. Moreover, alterations in miRNA expression profiles have been reported in neurodegenerative diseases, suggesting that miRNAs play a role in the neural and immune components of diseases. Therefore, identifying miRNAs that are dysregulated in all or most neurodegenerative diseases might contribute to the elucidation of common molecular mechanisms underlying neurodegeneration [47]. A previous study reported that the miRNAs that are most often dysregulated in neurodegenerative diseases are miR-9, miR-21, miR-29, miR-132, miR-124, miR-146a, miR-155, and miR-223 [47]. Furthermore, recent studies have identified mitochondrial DNA (mtDNA) as an interesting biomarker of neurodegenerative diseases; dysfunctions in the mitochondria and a low mtDNA copy number have been associated with the pathophysiology of neurodegenerative diseases [48]. Oxidative phosphorylation in the mitochondria generates reactive oxygen species, which play a key role in aging-related neurodegenerative diseases. Thus, epigenetic alterations, such as DNA methylation and hydroxymethylation, histone posttranslational modifications, and transcriptional dysregulations regulated by noncoding RNAs and miRNAs, might be involved in the development of neurodegenerative diseases, suggesting that mitochondrial oxidative phosphorylation is linked to alterations in miRNA expression [48].

#### 2.2.4. Involvement of miRNAs in Human Psychiatric Disorders

Although the underlying mechanisms of the involvement of miRNAs in human psychiatric disorders remain unclarified, recent studies have revealed altered miRNA expression profiles in the blood and brain of patients with psychiatric disorders, suggesting the potential of miRNA levels as a biomarker for the diagnosis of psychiatric disorders [49]. Recent research has indicated that miRNAs play a role in the control of N-methyl-D-aspartate receptor-dependent synaptic plasticity and psychiatric disorders [50].

#### 2.2.5. Involvement of miRNAs in Human Chronic Inflammatory Diseases

Various mechanisms regarding the role of miRNAs in human chronic inflammatory diseases have been investigated. These include (1) the switch between classically activated (M1) and alternatively activated (M2) macrophages in pulmonary diseases [51], (2) the involvement of miR-21 in the transforming growth factor-β1 (TGFβ1)-Smad3 signaling pathway in renal fibrosis and inflammation [52], and (3) the role of critical sets of miRNAs in chronic inflammation of the gastrointestinal tract, including inflammatory bowel diseases such as Crohn’s disease and ulcerative colitis [53]. In cells obtained from patients with inflammatory bowel diseases, the levels of multiple miRNAs, including miR-10, miR-21, miR-122, and miR-155, are significantly increased or decreased [54,55,56,57].

## 3. Non-Canonical isomiR Synthesis Pathway

**Figure 2:**
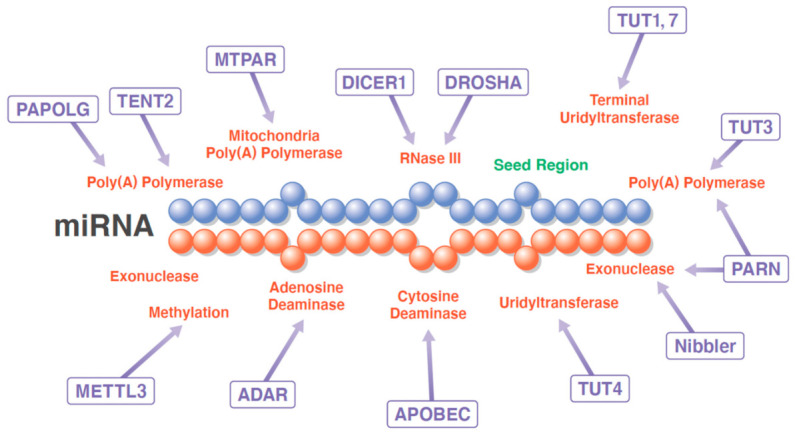
Generation of isomiR. Enzymes affecting the length, sequence, and modifications of miRNAs are: ADAR, adenosine deaminase RNA specific; APOBEC1, apolipoprotein B mRNA editing enzyme catalytic subunit; DICER1, dicer 1, ribonuclease III; DROSHA, drosha ribonuclease III; METTL3, methyltransferase-like protein 3; MTPAR, mitochondrial poly(A) polymerase; Nibbler, exonuclease 3′–5′ domain containing 3; PAPOLG, poly(A) polymerase gamma; PARN, poly(A)-specific ribonuclease; TENT2, terminal nucleotidyltransferase 2; TUT1, terminal uridylyltransferase 1; TUT3, terminal nucleotidyltransferase 4B; TUT4, terminal uridylyltransferase 4; and TUT7, terminal uridylyltransferase 7.

Although researchers analyzing tissues have classified miRNAs into canonical or non-canonical pathways, the classification based on their mechanism of action in nematodes has drawn considerable attention toward isomiRs. Owing to the development high-speed sequencing technology, isomiRs are being investigated and characterized as much as standard miRNAs, such as let-7. The rate of sequence difference in isomiRs is significantly lower in the 5′-terminus (5–15%) than in the 3′-terminus (40–50%) [58]. It has, also, been demonstrated that the 5′ isomiR-9-1 acquired the ability to inhibit the expression of DNA (cytosine-5-)-methyltransferase 3 beta (DNMT3B) and neural cell adhesion molecule 2 (NCAM2) but lost the ability to inhibit E-cadherin (CDH1), indicating that isomiRs of at least a small percentage of human miRNAs are the dominant transcript in certain cell types. Additionally, as indicated by the analysis of a miRNA database (miRBase), 5′ isomiRs have replaced canonical miRNAs several times during the course of evolution, suggesting their functional importance and contribution to the evolution of miRNA genes [58] (Figure 2).

### 3.1. Generation of Multiple Sequences from a Single miRNA Precursor

The central dogma of molecular biology states that the information of peptide-coding mRNAs reflects the sequence of genomic DNA, Thus, a miRNA precursor is considered to naturally generate miRNAs, with high fidelity. However, a recent study revealed that RNA editing events can result in greater variations of the genomic information, beyond what was previously considered as the physiological state, suggesting that this variability is further increased by the splicing of RNAs, albeit it occurs less frequently than the splicing of genomic sequences [59].

### 3.2. Enzymes Involved in isomiR Generation

Some enzymes affect miRNA length and sequence, leading to the synthesis of isomiRs, whereas other enzymes lead to chemical modifications of miRNAs, such as methylation, to form diverse groups of miRNAs called episomiRs.

#### 3.2.1. Enzymes Regulating miRNA Length

##### Poly(A)-Specific Ribonuclease (PARN)

PARN is a 3′-exoribonuclease with similarity to the RNase D family of 3′-exonucleases; PARN has a preference for poly(A) substrates and consequently degrades the poly(A) tails of mRNAs [60]. Although PARN is primarily involved in the exonucleolytic degradation of the poly(A) tail, which is the first step of eukaryotic mRNA decay, recent reports have shown that PARN controls the levels of several miRNAs. PARN loss-of-function mutations result in a severe form of hereditary disease, and previous research indicated that PARN deficiency affects not only the stability of noncoding RNAs, such as human telomerase RNA, but also the levels of several miRNAs in human cells [61]. Indeed, PARN regulates miRNA levels by recruiting the exonuclease DIS3, such as exosome 3′–5′ exoribonuclease (DIS3L) or DIS3L2, to degrade miRNAs in the cell [61]. PARN deficiency is, also, associated with an accumulation of p53 protein induced by the destabilization of miRNAs inhibiting translation [61] or by mRNA deadenylation [62], suggesting the significance of miRNA regulation by 3′-exoribonucleases. Another study indicated that the PARN-mediated shortening of miRNAs has little impact on their stability and is involved in the finalization of miRNA maturation, rather than the initiation of miRNA decay [63], suggesting that PARN-dependent 3′-end formation may be specific to several groups of miRNAs. A direct association of PARN with CUG-binding protein 1 (CUGBP1) has been shown to control the destabilization of miR-122, which plays a role in hepatocytes, by 3′ deadenylation [64], suggesting a UG-rich-sequence–specific mechanism.

##### Nibbler Homolog

Exonuclease 3′–5′ domain containing 3 (EXD3, Nibbler Homolog) is a protein with nucleic acid binding and 3′–5′ exonuclease activities. The 3′–5′ exonuclease activity is required for the catalytic 3′-end trimming of miRNAs and PIWI-interacting RNA (piRNA) biogenesis [65]. The N-terminal domain of Nibbler Homolog acts as a substrate recruitment platform for exonucleolytic miRNA maturation, achieved by the trimming of the 3′-end of miRNAs [66]. The significance of Nibbler Homolog in cancer remains to be fully elucidated.

##### Terminal Uridylyltransferase 1 (TUT1/TENT1)

The TUT1/TENT1 gene encodes a nucleotidyltransferase, which functions as a terminal uridylyltransferase, and a nuclear poly(A) polymerase, to generate mRNAs. Recent studies have shown the involvement of TUT1/TENT1 enzymatic activity in the modulation of miRNAs [67,68]. A study on osteosarcoma revealed that TUT1 inhibits the expression of peroxisome proliferator activated receptor gamma (PPARγ) and sterol regulatory element binding transcription factor 1 (SREBP-1c), which are critical for controlling lipogenesis by the upregulation of miRNA-24 and miRNA-29a, suggesting that TUT1 acts as a tumor suppressor in osteosarcoma [69].

##### Terminal Uridylyltransferase 7 or Zinc-Finger CCHC Domain-Containing Protein ([TUT7/TENT3B/ZCCHC6 or TUT4/TENT3A/ZCCHC11])

The TUT7/TENT3B/ZCCHC6 gene encodes a uridylyltransferase that mediates the terminal uridylation of mRNAs with poly(A) tails shorter than 25 nucleotides, to facilitate the global mRNA decay. Additionally, this enzyme is involved with TUT4/TENT3A/ZCCHC11 in miRNA-induced gene silencing, through the uridylation of deadenylated miRNA targets [70]. Uridylation by TUT7/TENT3B/ZCCHC6 regulates miRNA generation through three different mechanisms, indicating a dual role of uridylation, i.e., the repair and removal of defective pre-miRNAs [71]. TUT7/TENT3B/ZCCHC6, also, inhibits miRNA biogenesis, by mediating the terminal uridylation of miRNA precursors, including pre-let-7, preventing their processing by DICER and inducing their degradation. The degradation of pre-let-7 is strongly enhanced in the presence of Lin28, suggesting that Lin28 uses both TUT7/TENT3B/ZCCHC6 and TUT4/TENT3A/ZCCHC11 to control let-7 expression. This has important implications for stem cell biology and cancer [72].

##### Non-Canonical Poly(A) RNA Polymerase (TUT3/PAPD5/TENT4B)

TUT3/PAPD5/TENT4B gene encodes a terminal nucleotidyltransferase that, preferentially, catalyzes the transfer of ATP and GTP on 3′-poly(A) of RNAs leading to mRNAs stabilization. TUT3/PAPD5/TENT4B is the catalytic subunit of a TRAMP-like complex, with a poly(A) RNA polymerase activity that is involved in posttranscriptional quality-control mechanisms [73,74]. It has been reported that PAPD5 and PARN mediate the degradation of miR-21 through a tailing and trimming process, suggesting their implication in cancer and other proliferative diseases [75]. Moreover, human epidermal growth factor receptor 2 (HER2) signaling activates the transcription of mir-21, which is further stabilized by miR-4728-3p through the inhibition of TUT3/PAPD5/TENT4B [76], suggesting the significance of the non-canonical poly(A) polymerase TUT3/PAPD5/TENT4B pathway in cancer.

#### 3.2.2. Enzymes Determining miRNA Sequence

RNA editing is a regulatory mechanism that involves specific enzymes of the adenosine/cytidine deaminase family, which trigger single-nucleotide changes (A-to-I or C-to-U) within miRNA sequences, leading to the generation of different mature miRNAs with diverse functions [77]. Although the underlying mechanisms are not fully understood, RNA editing influences the stability, maturation, and activity of miRNAs, by changing their specificity toward targets. These changes might be involved in tumor heterogeneity and drug resistance. Thus, RNA editing may be beneficial for the design of new therapeutic approaches, based on miRNA targeting [77].

##### Adenosine Deaminases Acting on RNA (ADAR) Family

The ADAR proteins are, generally, much larger than the apolipoprotein B100 RNA editing catalytic subunit (APOBEC) proteins and contain one or more dsRNA-binding domains and one deaminase domain [78]. ADAR1 includes ADAR1p150, which has two Z-DNA binding domains, and ADAR1p110, which has one Z-DNA binding domain. ADAR1p150 and ADAR1p110 are produced from the same locus from different transcription initiation sites. The expression of ADAR1p150 is induced by interferon-α and β (type I IFN). Interestingly, ADAR1 has been, recently, identified as a suppressor of type I IFN signaling. Importantly, ADAR1 binds to pre-miRNAs and regulates their production. ADAR2 is strongly expressed in neural tissues and results in the RNA editing of GluA2, a subunit of the α-amino-3-hydroxy-5-methyl-4-isoxazole propionic acid (AMPA)-type glutamate receptor [78].

The ablation of ADARs has been reported to compromise pri-miRNA and pre-miRNA processing, resulting in altered miRNA levels [79]. Additionally, the destabilization of the miRNA structure, by editing events in regions outside the seed sequences, impairs the processing of miRNAs, including miR-151, let7-g, miR-33, miR-133a2, miR-197, miR-203, and miR-379, in the DROSHA/DICER complex [80,81], by altering the miRNA strand selection and loading on AGO [82,83].

##### APOBEC Family

Eleven types of APOBEC proteins are known in humans. They have one or two cytidine deaminase motifs [84]. APOBEC1 converts the cytosine in the apolipoprotein B100 mRNA to uracil in the small intestine and liver, generating a stop codon inside the translated region, which results in the conversion of mRNA that originally encodes ApoB100 protein into the mRNA encoding ApoB48 protein. ApoB48 plays different roles compared to ApoB100, in lipid transport. Thus, the APOBEC1 protein generates two types of proteins with different functions from one mRNA [84]. Activation-induced deaminase (AID) is a deaminase, which has been isolated after APOBEC1 and is responsible for gene reorganization phenomena, such as class switch recombination, somatic hypermutation, and gene conversion, which occur during antibody production [85]. The gene reorganization caused by AID leads to the diversification and affinity maturation of antibodies and is considered the key to acquired immunity [85].

Although many reports showed A-to-I substitution in miRNAs induced by ADAR proteins, the occurrence of C-to-U editing by members of the APOBEC family in miRNAs and its functional consequences on miRNA activity or specificity remain unclear. However, editing of 3′-UTRs of mRNAs by APOBEC proteins might significantly modify the functions of responsive elements to miRNAs [86,87].

#### 3.2.3. Enzymes Involved in miRNA Modifications

**Figure 3:**
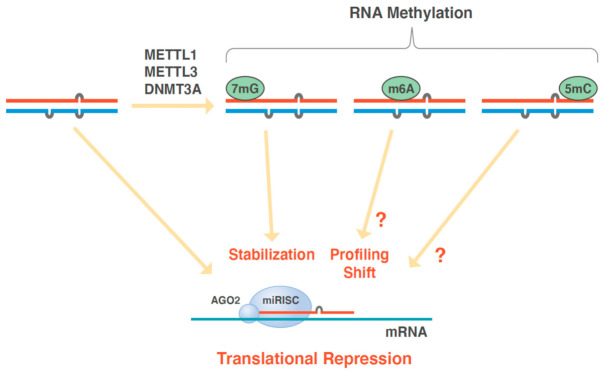
EpisomiR, a subtype of isomiR with different miRNA methylations. Although not fully understood, the methylation of miRNAs modulates miRNA silencing function and mRNA degradation. Recently developed state-of-the-art sequencing techniques, such as tunnel current sequencing and mass spectrometry analysis, enables the detection of m6A and 5mC modifications. 5mC, 5-methylcytosine; AGO2, argonaute RISC catalytic component 2; DNMT3A, DNA methyltransferase 3 alpha; m6A, N6-methyladenosine; m7G, 7-methylguanosine; METTL1, methyltransferase-like protein 1; METTL3, methyltransferase-like protein 3; miRISC, miRNA-induced silencing complex.

Although canonical isomiRs include, exclusively, the variation of the length and sequence of miRNAs, recent studies have indicated that miRNAs can harbor other variations, which are assumed to control their synthesis, stability, and translation in cells. Thus, the miRNA family is expanding [88]. In this article, we describe episomiRs as the miRNAs with sequence modifications that cannot be identified by standard methods, such as Sanger’s and Illumina sequencing, but which might be responsible for their functioning, similar to the role of tRNA modifications and metabolism in protein design [89]. The modifications of miRNAs include N6-methyladenosine (m6A) [90], 5-methylcytosine (5mC) [91], and 7-methylguanosine (m7G) [92]. It is well known that m7G is present in mRNA caps and at specific positions within tRNAs and ribosomal RNAs. Additionally, the m7G modification of tRNA has been proposed to be involved in the translational regulation of the development of intrahepatic cholangiocarcinoma [93]. Further, N 7-methylguanosine tRNA modification has been shown to enhance oncogenic mRNA translation and promote cancer progression [94]. The significance of episomiRs was noted in gastrointestinal cancer [90,91,92,93,94]. Given that a recent study has indicated that methyltransferase 1 (METTL1) promotes miRNA processing through m7G [92], the importance of profiling miRNA modifications has emerged (Figure 3; see, also, the following section).

##### Functions of EpisomiR

Methyltransferase 4 (METTL4) adds m6A to small nuclear RNAs and is, predominantly, responsible for the increase in m6A modifications of vasoactive miRNAs, which are expressed in primary human fibroblasts and human fibroblast cell line under hypoxic conditions, and these m6A-modified miRNAs directly affect the efficacy of repressing downstream mRNA targets [95]. It is not, completely, understood at what point miRNAs are methylated, i.e., at the pri-miRNA, pre-miRNA, or mature miRNA stage; however, mature miRNAs are too short to be acted upon by epitranscriptomic enzymes. Modifications of long noncoding RNAs are well known in cancer research and are reviewed elsewhere [96]. A recent study has indicated that METTL3 accelerates miR-150 maturation by mediating m6A methylation of pri-miR-150 at locus 498, through cooperation with the “m6A reader” YTH N6-methyladenosine RNA-binding protein 2 (YTHDF2). This methylation plays a role in miR-150 targeting brain-derived neurotrophic factor (BDNF) mRNA, and METTL3/miR-150/BDNF is a regulatory pathway in neuropathic pain, induced by a lesion to the somatosensory nervous system [97]. A study on miR-21-5p, an oncogenic miRNA, showed the highest m6A enrichment in human non-small-cell lung cancer cells. Additionally, the m6A marks in mature miR-21-5p might, directly, affect its silencing potential toward target genes and impair its effect of promoting proliferation and motility. Thus, episomiRs have novel functions induced by the methylation of miRNAs [98].

As for m6A and 5mC modifications, the preceding modification, possibly, affects subsequent modifications, and these modifications influence each other. The phenomenon might be used to improve the accuracy and speed of modification detection [91]. Moreover, a previous study indicated that increased m7G modifications of a subset of tRNAs by the METTL1/tRNA (guanine-N(7)-)-methyltransferase non-catalytic subunit (WDR4) complex stabilizes mRNAs, increases the translation efficiency, reduces ribosome pausing, and is associated with poor survival in human cancers [93]. Furthermore, it has been demonstrated that METTL1-mediated methylation augments the processing of the miRNA let-7, by disrupting an inhibitory secondary structure within the pri-miRNA. This suggests that METTL1-dependent N7-methylation of guanosine plays a role in cell migration, by regulating miRNA structure and biogenesis [92].

##### Identification of EpisomiR

To obtain information on RNA modifications of episomiR, several methods have been applied [99] (Table 1). RNA immunoprecipitation sequencing locates sites, where proteins bind to RNAs with m6A modifications. This technique might provide high-throughput information regarding the modifications in episomiRs, although a single-nucleotide resolution cannot be achieved. Additionally, capture mass spectrometry (capture MS) methods are useful, for studying methylation sites at the single-nucleotide level [90]. Given that the throughput of capture MS methods is limited, a recent study indicated that the usage of quantum sequencer might be complementary and beneficial for the direct detection of methylation sites, such as m6A and 5mC, in episomiRs [91]. This state-of-the-art technology is expected to enable the profiling of previously uncharacterized molecular targets, which will be indispensable for the precise diagnosis of diseases and the development of innovative therapeutic approaches. The technology can not only detect m6A, 5mC, and m7G modifications but also, if optimized, enable the detection of yet uncharacterized modifications. Furthermore, with the recent development of technologies, such as high-throughput sequencing, m6A, 5mC, and m7G modifications can be analyzed at a single-cell level, thereby expanding the knowledge of episomiR and its role in human diseases [90,91,92,93,94,100,101,102].

##### Sequencing of miRNAs in Single Cells

Notably, recent studies have sequenced RNA modifications using little amounts of samples. A new technology, called “deamination adjacent to RNA modification targets” (DART-seq), was developed for transcriptome-wide m6A mapping, by utilizing a fusion protein consisting of the m6A-binding YTH domain tethered to the cytidine deaminase APOBEC1 to direct C-to-U editing at cytidine residues that invariably follow m6A sites [100,101]. Another study described m6A-SAC-seq, which consists of m6A-selective allyl chemical labeling and sequencing, as a method for quantitative, whole-transcriptome mapping of m6A at a single-nucleotide resolution [102]. However, sequencing miRNAs at a single-cell level requires further optimization. On the other hand, a study on tRNA demonstrated the crucial role of 7mG, an important modification in the cap site of mRNA, both in oncogenic transformation [94,103,104] and in the processing of miRNAs controlling the expression of the high mobility group AT-hook 2 (HMGA2) gene [92]. In particular, m7G promotes the processing of miRNA precursors, by antagonizing G-quadruplex structures [92,105]. These technologies, using chemical modifications, need to be further developed to enable the use of small sample amounts and single-cell resolution.

## 4. Conclusions

**Figure 4:**
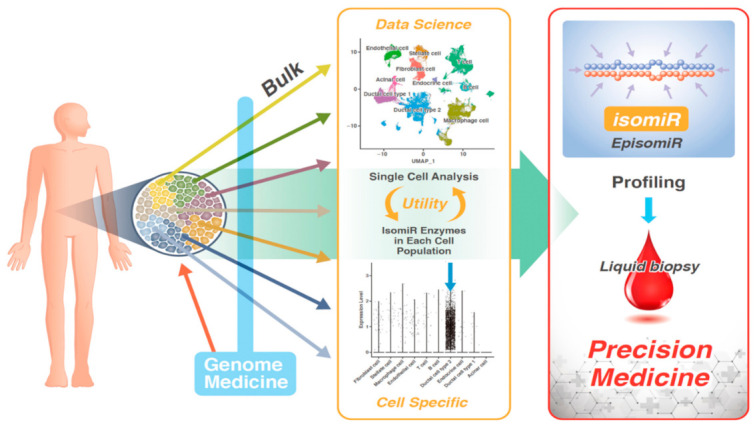
Outlook on high-precision medical care, achieved by the profiling of isomiR. A recent study, using single-cell sequencing of various tissues, has provided links with data from liquid biopsy, such as that of peripheral blood. Given that the expression of isomiR-generating enzymes varies, according to the cell populations present in tissues, the study of isomiR will enable the detection of cell-specific expression patterns. This will improve the diagnosis of diseases, in which unique expression patterns are observed due to genomic and epigenomic variabilities, and will enable the development of precision medicine based on liquid biopsy analyses.

Since cancer is a genetic disease, the study of genomic alterations in human cancer has enabled the identification of the miRNAs in cancers [38,39]. Since these pioneering discoveries, the existence of miRNA variants, called isomiRs, which differ in length and sequences, have been revealed over two decades ago. Additionally, another subbranch of the isomiR family, called episomiR, has, now, emerged and comprises miRNA variants with different modifications (Table 2). Since the heterogeneity of tumors reflects the diversity of mechanisms involved in the occurrence and development of cancer, elucidating the precise miRNA alterations and compiling bioinformatics data from transcriptomic analyses is expected to contribute to the development of precision medicine against cancer (Figure 4).

## Figures and Tables

**Table 1:** Sequencing techniques for the investigation of episomiRs.

Technology	Target (RNA)	Single-Cell Level	Single Molecule Level	Throughput	Reference
Cap-MS	m6A, 5mC, and others (miR)	n.d.	possible	low	[90]
Tunnel current sequencing	m6A, 5mC, and others (miR)	n.d.	applicable	moderate or high	[91]
TRAC-seq	7mG (tRNA; the technology can be possibly applied to miR)	possible	possible	high	[94]
BoRed-seq	7mG (miR)	possible	possible	high	[92]
scDART-seq	m6A (mRNA, possible to miR)	applicable	possible	high	[101]
M6A-SAC-seq	m6A (mRNA, possible to miR)	applicable	applicable	high	[102]

BoRed-seq, borohydride reduction sequencing; Cap-MS, captured mass spectrometry; DART-seq, single-cell deamination adjacent to RNA modification targets; miR, microRNA; m6A-SAC-seq, m6A-selective allyl chemical labeling and sequencing; n.d., not yet determined; TRAC-seq, m7G site-specific tRNA reduction and cleavage sequencing.

**Table 2:** IsomiR-related genes and human diseases.

isomiR-Related Genes	Human Diseases	Reference
*DICER1*	pleuropulmonary blastoma familial tumor predisposition syndrome (G)	[106]
*DROSHA*	ovarian cancer (S)	[107]
*TUT1*	retinitis pigmentosa (U)	[108]
*TUT4, TUT7*	Perlman syndrome (U)	[109]
*PARN*	dyskeratosis congenita (G)	[110]
*ADAR*	dyschromatosis symmetrica hereditaria (G)	[111]
*APOBEC*	bladder cancer (U)	[112]
*METTL3*	acute myeloid leukemia (S)	[113]

G, germline mutations; S, somatic mutations; U, unknown.

## Data Availability

Not applicable.
